# Malaria vectors and transmission dynamics in coastal south-western Cameroon

**DOI:** 10.1186/1475-2875-6-5

**Published:** 2007-01-17

**Authors:** Jude D Bigoga, Lucien Manga, Vincent PK Titanji, Maureen Coetzee, Rose GF Leke

**Affiliations:** 1 Department of Biochemistry, Faculty of Science, University of Yaounde I, Cameroon; 2 The Biotechnology Center, University of Yaounde I, P.O. Box 3851-Messa, Yaounde, Cameroon; 3 Vector Biology and Control Unit, WHO Regional Office for Africa, Brazzaville, Congo; 4 Department of Life Sciences, Faculty of Science, University of Buea, Cameroon; 5 Vector Control Reference Unit, National Institute for Communicable Diseases, NHLS, Johannesburg, South Africa; 6 Division of Virology and Communicable Disease Surveillance, School of Pathology of the National Health Laboratory Service and the University of the Witwatersrand, Johannesburg, South Africa; 7 Faculty of Medicine and Biomedical Sciences, University of Yaounde I, Cameroon

## Abstract

**Background:**

Malaria is a major public health problem in Cameroon. Unlike in the southern forested areas where the epidemiology of malaria has been better studied prior to the implementation of control activities, little is known about the distribution and role of anophelines in malaria transmission in the coastal areas.

**Methods:**

A 12-month longitudinal entomological survey was conducted in Tiko, Limbe and Idenau from August 2001 to July 2002. Mosquitoes captured indoors on human volunteers were identified morphologically. Species of the *Anopheles gambiae *complex were identified using the polymerase chain reaction (PCR). Mosquito infectivity was detected by the enzyme-linked immunosorbent assay and PCR. Malariometric indices (plasmodic index, gametocytic index, parasite species prevalence) were determined in three age groups (<5 yrs, 5–15 yrs, >15 yrs) and followed-up once every three months.

**Results:**

In all, 2,773 malaria vectors comprising *Anopheles gambiae *(78.2%), *Anopheles funestus *(17.4%) and *Anopheles nili *(7.4%) were captured. *Anopheles melas *was not anthropophagic. *Anopheles gambiae *had the highest infection rates. There were 287, 160 and 149 infective bites/person/year in Tiko, Limbe and Idenau, respectively. *Anopheles gambiae *accounted for 72.7%, *An. funestus *for 23% and *An. nili *for 4.3% of the transmission. The prevalence of malaria parasitaemia was 41.5% in children <5 years of age, 31.5% in those 5–15 years and 10.5% in those >15 years, and *Plasmodium falciparum *was the predominant parasite species.

**Conclusion:**

Malaria transmission is perennial, rainfall dependent and *An. melas *does not contribute to transmission. These findings are important in the planning and implementation of malaria control activities in coastal Cameroon and West Africa.

## Background

Malaria is a major public health problem in Cameroon [1-3]. Over 900,000 clinical cases occur yearly and are responsible for 40–45% hospital consultations, 20% hospital admissions and 35–40% deaths. Children less than five years old are the most affected [4]. Despite efforts made by the National Malaria Control Programme to curb the disease burden, the prevalence is seemingly on the increase. Previous studies have attributed this to the increasing spread of drug resistance in the parasite, insecticide resistance in the vectors, inadequate and inconsistent allocation of resources for control [5,6] and the presence of very efficient mosquito vectors of *Plasmodium falciparum *[7-9].

Malaria vector control activities in Cameroon focus mainly on the use of insecticide-treated bed nets. However, the implementation of effective vector control strategies requires requisite information on the vector population structure, their distribution and efficiency in malaria transmission. Previous studies in Cameroon have shown that the intensity and duration of transmission, as well as the vector species, vary greatly between different eco-zones of the country, from perennial transmission in the southern forested regions to seasonal and unstable transmission in the northern Sudano-savannah and Sahelian savannah regions [8-13]. At least 14 out of the 45 species of *Anopheles *described in Cameroon are capable of transmitting malaria. The most common and efficient vector species are *Anopheles gambiae, Anopheles. arabiensis Anopheles funestus, Anopheles nili *and *Anopheles moucheti *[14]. Species such as *Anopheles paludis*, *Anopheles pharoensis *and *Anopheles hankocki *play only minor, secondary roles in malaria transmission [8,15].

Although *Anopheles melas*, a member of the *An. gambiae *complex, is purported to be an important malaria vector along the coast of many West African countries, its importance in malaria transmission in Cameroon is not clear. Although few studies have reported on the occurrence of *An. melas *in Cameroon [16], currently there is insufficient information on its abundance and infection rates. This may be due, in part, to the fact that early studies on *An. gambiae s.l. *relied on morphological identification only and more recent studies have not used modern methods like PCR because they are expensive and expertise demanding.

Unlike in the southern forested and northern savannah regions of Cameroon, where the epidemiology of malaria has been better studied prior to the implementation of malaria control activities, very little is known about the vectors and their contribution to malaria transmission in the coastal areas. The coastal area of south-west Cameroon is a characteristic ecological area. It has undergone serious environmental modifications over the years owing to rapid growth in population, urbanization and the agro-industrial activities of the Cameroon Development Corporation (CDC), the largest agricultural scheme in central Africa. Such modifications can lead to ecological changes that affect the vector population structure, distribution and density, all of which would impact on the efficiency in transmitting malaria. Thus, this paper describes the anopheline species composition and malaria transmission dynamics in three localities (Tiko, Limbe and Idenau) along the Atlantic coast of south-western Cameroon.

## Methods

### Description of the study area

The study was conducted in Tiko (Small Ikange) 4° 04'N, 9°17'E – a rural setting; Limbe (Middle Farm) 4°02'N, 9°11'E – a cosmopolitan town; and Idenau (Bibunde-Scipio) 4°01'N, 9°03'E – a semi-urban vicinity situated along the Atlantic coast of Cameroon. Idenau borders the Atlantic Ocean; while Tiko is situated 20 km from Limbe, 50 km from Idenau and 19 km from the coastline. Limbe is situated 30 km from Idenau and 8 km from the coastline. The area lies within the tropical rain forest region of Central Africa and harbours the largest agro-industrial scheme in the country and sub region, the Cameroon Development Corporation (CDC). Crops grown in the plantations include rubber (*Fiscus elastica*) and banana (*Musa spp.*) in Tiko, and oil palm (*Elaies Spp.*) in Tiko, Limbe and Idenau. Plantation workers are housed in camps with few medical facilities at their disposal. The population is highly heterogeneous comprising people from almost every ethnic community in Cameroon, some parts of neighbouring Nigeria, Niger and Ghana. The climate is typically equatorial with a mean annual temperature of 26°C, mean annual rainfall of 2,000–10,000 mm and 88% relative humidity. There are two seasons; the dry season from November to February and the wet season from March to October.

### Study design and ethical considerations

A longitudinal study was carried out for 12 months from August 2001 to July 2002. Permission to carry out this study was obtained from the Human Resources and Health departments of the Cameroon Development Corporation (CDC), and an ethical clearance obtained from the National Ethical Review Committee of Cameroon. A sensitization rally was organized with the population during which the purpose of the study was clearly explained. Participation in the study was voluntary. All diagnosed cases of malaria were treated for free, following recommendations set by the national malaria control program.

### Collection and processing of adult mosquitoes specimens

For two consecutive nights (6:00 pm–6:00 am) every month, at each site (total 72 nights), mosquitoes were captured indoors on six human volunteer collectors using the human landing catch method. These were sorted and the anophelines morphologically identified to groups using the taxonomic keys of Gillies and De Meillon [17] and Gillies and Coetzee [18]. The ovaries of a random sample of unfed specimens were extracted and examined for parity determination [19]. The carcasses of each dissected and those undissected mosquitoes were individually preserved on cotton wool over a desiccant (silica gel) in labeled tubes for the necessary immunological and molecular biologic analyses.

### Larval collection, rearing and processing of adult *Anopheles gambiae*

*Anopheles gambiae *larvae were collected from potential breeding sources by dipping. The larvae were graded into different breeding containers according to whether they were in their first, second, third or fourth instars. The larvae were fed with finely ground dog biscuit until the adults emerged. The emerging adults were maintained on 10% sugar solution until identified to species level by polymerase chain reaction (PCR) as described by Scott et al. [20].

### PCR identification of the *Anopheles gambiae *complex

In cases where the number of *An. gambiae *mosquitoes collected per month exceeded 40, the legs and wings of at least 40 were used for species identification by the polymerase chain reaction method [20]. Positive control mosquitoes for *An. melas*, *An. gambiae s.s.*, *An. arabiensis *and *Anopheles quadriannulatus *(obtained from the National Institute for Communicable Diseases, Johannesburg, South Africa) and negative controls (tubes containing all the components of a PCR reaction mixture but without template DNA) were assayed simultaneously. *Anopheles gambiae *specimens were identified to molecular M or S form as described by Favia and others [21].

### ELISA for detection and PCR for confirmation of parasite infections in mosquitoes

The head and thorax of each mosquito were separated from the rest of the body, homogenized in blocking buffer (0.5%Casein, 0.1 N NaOH, 1 × PBS) and a portion of the homogenate assayed by ELISA for the presence of circumsporozoite antigens (CSA) of *P. falciparum*, *Plasmodium malariae*, *Plasmodium ovale *and two *Plasmodium vivax *strains (Pv210 and Pv247) [22,23]. Positive controls (Kikergaard & Perry Laboratories, USA) and negative controls (uninfected laboratory reared mosquitoes) were assayed simultaneously. A specimen was considered positive if a visual green colour was detected with an OD value (at 405 nm) of at least the mean of the negative controls plus two standard deviations [24]. *Plasmodium *DNA was extracted from the remainder of the head-thorax homogenate of all ELISA positive specimens using phenol and examined for sporozoites by PCR [25,26]. To minimize having false positive ELISA [27,28] only PCR confirmed specimens were used to determine the infection rates. Sporozoite densities and Sporozoite antigen equivalencies were estimated using standard graphs for estimating sporozoite numbers from absorbance values provided with the ELISA kit.

### Malaria prevalence survey

Malaria prevalence surveys were conducted four times in the human population: in August 2001 (rainy season), November 2001 (transition: rainy to dry season), February 2002 (dry season) and May 2002 (transition: dry to rainy season), in a cohort of subjects from thirty randomly selected homes at each study site. Thick and thin blood smears were made using finger prick blood samples. The blood smears were fixed in methanol (thin smears only), and stained with Diff-quick stain (Baxter International, Deerfield, IL, USA). The thick smears were independently examined for the presence of malaria parasites by two microscopists (unaware of each other's results). An individual was considered positive if malaria parasites were detected in the blood smear. A blood smear was considered negative if parasites were not detected after examining 200 oil-immersion fields of the thick smear. When malaria parasites were detected in a blood film, the parasite density was determined by counting the number of parasites present per 200 white blood cells in a thick smear and multiplying by 40 to arrive at an approximate parasite count per microlitre of blood. This was based on the assumption that the average WBC count was 8,000/μl blood [29]. The gametocyte index was calculated as the proportion of positive slides containing gametocytes after examining 200 oil-immersion fields. The thin smears were examined to identify the *Plasmodium *species. However, species identification was further confirmed by PCR, using *Plasmodium *DNA isolated by the chelex method from a portion of each blood sample collected on filter paper [30].

### Data analysis

The total number of mosquitoes collected, irrespective of the species, was used to assess the level of nuisance to man. Entomological parameters considered were: 1) Man-biting rate (ma), calculated as the average number of bites received per person per night of collection; 2) Infection rate, measured as the proportion of mosquitoes found to contain circum-sporozoite antigen by ELISA. 3) Parity rate, measured as the ratio of parous mosquitoes to the sum total of parous and nulliparous mosquitoes dissected. 4) Entomological Inoculation Rate (EIR), which was calculated as the product of the man biting rate and circumsporozoite antigen rate as confirmed by PCR. Sporozoite densities and Sporozoite antigen equivalencies were estimated using standard graphs provided with the ELISA kit. Chi-square statistics was used to compare the number of mosquitoes at different sites and CSA rates.

## Results

### Composition of mosquito species biting humans

**Figure 1:**
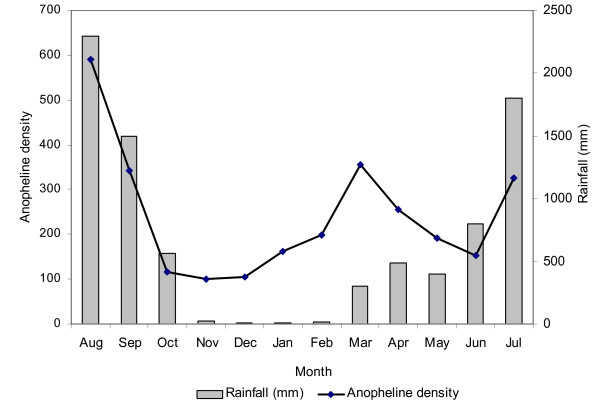
Monthly variation in the density of anopheline vectors of human malaria (line) in relation to rainfall (bars).

A total of 3,852 mosquitoes were collected during 432 human nights at the three localities. The species found were *An. gambiae *(57%), *An. funestus *(10%) and *An. nili *(5%) as the malaria vectors, and *Anopheles rufipes *(9%), *Culex *spp. (17%) and *Mansonia *spp. (2%) as non-malaria vectors. Table 1 shows the distribution of mosquito species by locality. Amongst the 2,773 female *Anopheles *vectors of malaria collected 79% were *An. gambiae*, 14% *An. funestus *and 7% *An. nili*. The density of anopheline species followed trends in rainfall patterns, peaking during periods of heavy rainfall especially between July and September (Figure 1).

**Table 1:** Distribution of the mosquito populations biting humans by study locality in coastal Cameroon.

	Mosquito species	Tiko	Limbe	Idenau	Total Collected
Malaria vectors	*Anopheles gambiae*	1086 (51%)	619 (56.5%)	463 (74.5%)	2168
	*Anopheles funestus*	305 (14%)	79 (7.2%)	15 (2.5%)	399
	*Anopheles nili*	205 (10%)	1	-	206
Non-malaria vectors	*Anopheles rufipes*	334 (16%)	23 (2%)	-	357
	*Culex sp.*	135 (6%)	365 (33.3%)	135 (21.7%)	635
	*Mansonia sp.*	70 (3%)	9 (1%)	8 (1.3%)	87

	**Total**	**2135(100%)**	**1096 (100%)**	**621 (100%)**	**3852**

Out of the 2,168 *An. gambiae *collected feeding on humans, all 1,240 identified by PCR were *An. gambiae s.s. *and only the M molecular form was detected. No *An. melas *was identified amongst specimens caught biting humans at all three localities. Adult *An. gambiae *complex mosquitoes bred from larval collections were identified as *An. gambiae s.s*. M form (85.6%) and *An. melas *(14.4%) with the latter species found only in Limbe and Idenau (Table 2).

**Table 2:** Distribution of *Anopheles gambiae *complex adults caught feeding on humans and bred from larval collections in Tiko, Limbe and Idenau, coastal Cameroon.

	Adult *Anopheles gambiae *feeding on humans				*An gambiae *bred from larval collections			
	
Locality	Total collected	Total examined by PCR	*Anopheles gambiae *s.s		Total examined By PCR	*Anopheles gambiae s.s.*		*Anopheles melas*
						
			M form	S form		M form	S form	
Tiko	1086	540	540 (100%)	0	60	60 (100%)	0	0
Limbe	619	400	400 (100%)	0	60	52 (86.7%)	0	8(13.3%)
Idenau	463	300	300 (100%)	0	60	42 (70%)	0	18(30%)

**Total**	**2168**	**1240**	**1240 (100%)**	**0**	180	154 (85.6%)	0	26 (14.6%)

### Parity rates

Table 3 gives the parity rates of the malaria vectors in the three localities. The overall parity rate was 64.2% (n = 1520). Generally, *An. gambiae *had very high parity rates at all the localities, although there were more parous *An. nili *in Tiko (75.8%). The parity rates were observably higher during the dry season with peak monthly feeding for the parous mosquitoes consistently between 24:00 and 02:00 hours. The number of *An. funestus *mosquitoes collected in Limbe and Idenau was too small for statistical analysis.

**Table 3:** Parity rates of the malaria vectors at the three study sites.

**Study site**	**Total No. Dissected**	**Species**	**Parity rate**
Tiko	823	*An. gambiae*	65.2% (n = 528)
		*An. funestus*	54.3% (n = 175)
		*An. nili*	75.8% (n = 120)
Limbe	397	*An. gambiae*	63.8% (n = 351)
		*An. funestus*	56.5% (n = 46)
Idenau	300	*An. gambiae*	69.4% (n = 285)
		*An. funestus*	53.3% (n = 15)

### Biting rates and seasonality

**Figure 2:**
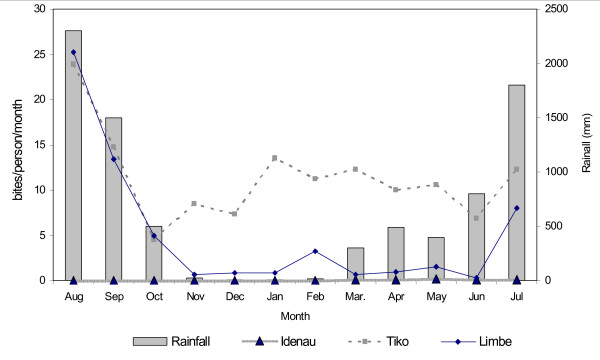
Relationship between rainfall and biting rate of the vectors in the three localities.

The mean daily biting rate was highest for *An. gambiae *(Table 4). Where *An. nili *was found, particularly in Tiko, peak biting was in May, while *An. funestus *peaked in March. The mean daily biting rates (bites/person/night) varied seasonally, increasing with the amount of rainfall (Figure 2). The peak bite rate was recorded during August, in the peak of the rainy season. The frequency of human attack at night was observed to increase gradually between 20:00 and 22:00 hours, then peaking between 22:00 and 02:00 hours and then declining speedily towards dawn. The biting rate was generally higher in Tiko.

**Table 4:** Mean daily man biting rates (ma), circumsporozoite antigen rates (CSA) and entomological inoculation rates (EIR – infective bites per person per year) of the malaria vectors by locality in coastal Cameroon.

Locality		*Anopheles gambiae*	*Anopheles funestus*	*Anopheles nili*
Tiko	Total collected	1086	305	205
	*ma*	7.5	2.1	1.4
	Tested for CSP	1086	305	205
	CSA rate	7.1 ± 1.5%	17 ± 4.2%	6.3 ± 3.3%
	EIR [95% CI]	177 [161.0–193.1]	85 [69.8–100.1]	24 [14.8–33.2]
Limbe	Total collected	619	79	-
	*ma*	4.3	0.6	-
	Tested for CSP	619	79	-
	CSA rate	8.4 ± 2.0%	11 ± 7.0%	-
	EIR [95% CI]	133 [123.7–142.2]	27 [22.4–31.68]	-
Idenau	Total collected	463	15	-
	*ma*	3.2	0.1	-
	Tested for CSP	463	15	-
	CSA rate	10.8 ± 2.8%	10/15	-
	EIR [95% CI]	124 [115.0–132.9]	25 [16.9–33.9]	-

### Vector infection rates

**Figure 3:**
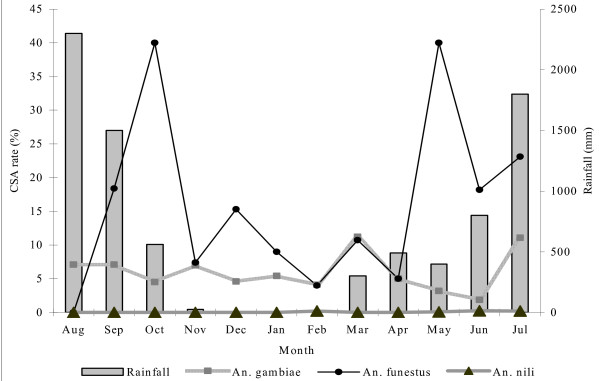
Combined monthly circumsporozoite antigen rates for *Anopheles gambiae, An. funestus *and *An. nili *in relation to rainfall in Tiko, Limbe and Idenau.

Details of the mean CSA rates and monthly variation by locality and by vector species are shown in Table 4 and Figure 3. When the 2,773 female *Anopheles *mosquitoes were examined by ELISA only, 298 (10.7%) were found to be infected. Following PCR on the positive ELISA specimens, 263 (9.48%) out of the 298 were positive for either *P. falciparum *or *P. malariae *or both (88.3% confirmation rate). Of these, there were 8.3% infections in *An. gambiae*, 17.8% in *An. funestus *and 6.3% in *An. nili*. Generally, *An. funestus *had higher CSA rates compared with *An. gambiae *and *An. nili*, where they co-existed. CSA rates of up to 40% were recorded between June and October, during the rainy season.

Of the 263 PCR-confirmed infections detected in the vectors, 90% were *P. falciparum *and 10% *P. malariae*. Neither *P. ovale *nor *P. vivax *infections were found in the mosquitoes. Only 5% of the mosquitoes (all *An. gambiae*) carried mixed *Plasmodium *infections. There were more infected *An. gambiae *during the wet season, maintaining low but similar proportions throughout the other months of the year. The estimated mean sporozoite density for all positive anophelines was 1,200 for *P*. *falciparum *infections and 400 for *P. malariae*, while the antigen equivalence was 37.5 pg for *P*. *falciparum *and 12.5 pg for *P*. *malariae*.

In Tiko, 142 infections were detected, 77 in *An. gambiae *(91% *P. falciparum *and 9% *P. malariae*), 52 in *An. funestus *(92.3% *P. falciparum *and 7.7% *P. malariae*) and 13 in *An. nili *(all *P. falciparum*). No cases of mixed infections were found. In Limbe, there were 8.4% (n = 619) infections in *An. gambiae*, 11.4% (n = 79) in *An. funestus *and none in *An. nili*. The highest number of infections (28.5%) was recorded in January and in April. Infected *An. funestus *were found only during three months (September, January and July). The difference between the CSA rates for *An. funestus *and *An. gambiae *in Limbe was not significant (p = 0.3). In Idenau, there were 10.8% (n = 463) infections in *An. gambiae *and 10/15 in *An. funestus*. Infections in *An. gambiae *were found only during the rainy season, increasing steadily from the onset of the rains and peaking in September (33.3%).

### Entomological inoculation rates (EIR)

**Figure 4:**
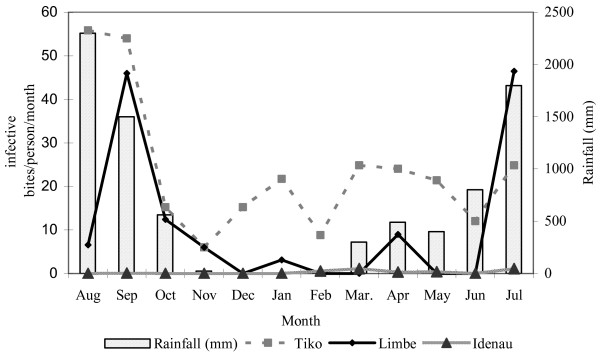
Combined monthly Entomological Inoculation Rates for the vector species in Tiko, Limbe and Idenau.

Details of the number of infective bites/person/year (ib/p/y) by vector species in the three localities are presented in Table 4. Figure 4 shows the monthly variation in the combined EIRs for the vector species in the surveyed areas. The annual EIR in Tiko was 287 ib/p/y, where *An. gambiae *was the main vector species and was responsible for 177 [95%CI: 161.0–193.1] ib/p/y. It contributed to transmission every month of the year and more intensely during the rainy season, with a peak in August (56 ib/p/m). *Anopheles funestus *and *An. nili *were the secondary vectors, with *An. funestus *transmitting malaria only during the rainy season with a peak in September (33 ib/p/m). In Limbe and Idenau, where *An. gambiae *and *An. funestus *were the only vectors, *An. gambiae *was predominant and responsible for 133/160 [95%CI: 123.7–142.2] ib/p/y in Limbe and for 124/149 [95%CI: 115.0–132.9] ib/p/y in Idenau. Peak transmission in Idenau was in July, during the rainy season.

### Malaria prevalence survey

#### Plasmodic index (PI)

**Figure 5:**
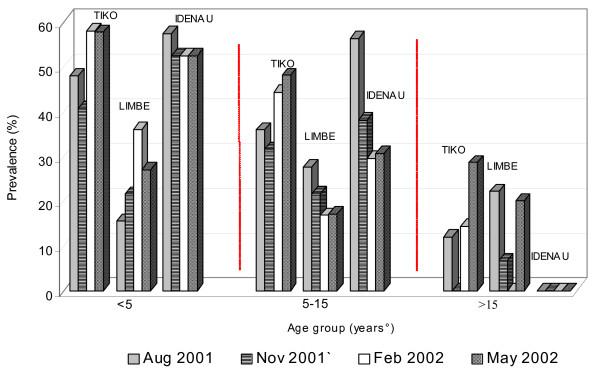
Prevalence of malaria parasitaemia by age group and season in Tiko, Limbe and Idenau.

The prevalence by age group was 41.5% (n = 595) for those less than 5 years of age, 31.5% (n = 923) for those 5–15 years old and 10.5% (n = 172) for those older than 15 years (Figure 5). The plasmodic index in Tiko was 39% (n = 587) and distributed as 50.7%, 40.4% and 12% for the age groups <5 years, 5–15 years and >15 years respectively. The highest number of cases (67/164) was recorded in May, in the early parts of the rainy season and least in November (38/126), the transition from rainy to dry season. The plasmodic index in February was 44.9% (n = 127) and in August 36% (n = 200). A significant difference was observed in the between season plasmodic indices (p = 0.04).

The plasmodic index in Limbe was 25.3% (n = 689) distributed as 22.2%, 21.5% and 15% in the <5 years, 5 – 15 years > 15 years respectively. The prevalence during each sampling period was 22.6% (n = 208) in August, 20.6% (n = 247) in November, 20.9% (n = 116) in February and 21.2% (25/118) in May. The difference between sampling periods was not significant (p = 0.9). The prevalence in Idenau was 42.7% (n = 174). The most affected were the age group < 5 years old with a prevalence of 54%. The age group 5–15 years had a prevalence of 39% (n = 213). No infections were found in persons older than 15 years.

#### Parasite species

Infections were detected as either single infections of *P. falciparum *only, mixed infections of *P. falciparum *and *P. malariae *or a cocktail of *P. falciparum*,*P. malariae *and *P. ovale. Plasmodium falciparum *alone accounted for 85% of all infections detected. There were 14% cases of mixed infections of *P. falciparum *and *P. malariae *and 1% mixed infections of the three parasites.

A total of 121 infected cases were detected in Tiko with 83.5% single infections of only *P. falciparum*, 11.6% mixed *P. falciparum *and *P. malariae *infections and 4.9% triple infections of *P. falciparum*, *P. malariae *and *P. ovale*. At least 50% of all cases in Tiko during each sampling period were children less than five years old. In Limbe, 88% (n = 110) of the infections were *P. falciparum *alone. There were, 10% mixed infections of *P. falciparum *and *P. malariae*, and 1.8% mixed infections of *P. falciparum*,*P. malariae *and *P. ovale*. A total of 129 infected cases were detected in Idenau of which 92.2% were single infections of *P. falciparum*, 6.2% mixed infections of *P. falciparum *and *P. malariae *and 1.4% mixed infections of *P. falciparum, P. malariae *and *P. ovale*.

#### Gametocyte index

The mean gametocyte index was 2.7% (n = 1691). All gametocytes were identified as *P. falciparum *and mostly in children less than five years of age. Table 5 shows the distribution of the mean gametocyte index at the three localities.

**Table 5:** Distribution of the mean gametocyte index in children under 5 years by locality in coastal Cameroon.

**Season**	**Locality**			**Total**
		
	**Tiko**	**Limbe**	**Idenau**	
August 2001 (Rainy Season)	4/200 (2.0 ± 0.1%)	3/208 (1.4 ± 0.1%)	4/120 (3.3 ± 0.1%)	11/528 (2.1%)
November 2001 (Transition:Rainy-dry season)	4/134 (3.0 ± 0.1%)	3/118 (2.5 ± 0.1%)	6/95 (6.3 ± 0.1%)	13/347 (3.7%)
February 2002 (Dry season)	5/127 (4.0 ± 0.1%)	2/116 (1.7 ± 0.1%)	3/94 (3.2 ± 0.1%)	10/337 (3.0%)
May 2002 (Transition: dry-Rainy season)	3/126 (2.4 ± 0.1%)	3/247 (1.2 ± 0.1%)	4/106 (3.8 ± 0.1%)	10/479 (2.1%)

#### Parasite density

Slide examination was done independently by two microscopists and the mean parasite density considered. Where the difference in parasite density was more than 20 parasites/μl, the slide was read by a third person and the mean of the two nearest values considered. In Tiko, the mean parasite count throughout the four sampling periods was 6,035 parasites/μl of blood. The highest count (103,000/μl) was determined in a two-year old in November, the end of the rains. In Limbe, the mean parasite count during the four sampling periods was 6,103/μl of blood and the highest count (90,000/μl) was detected in a one-year-old in May, during the rainy season. The mean parasite count in Idenau was 4,098/μl with the highest count observed in a one year old in May (56,000/μl) at the onset of the rains.

## Discussion

The findings of this study show clearly that malaria transmission in coastal south-west Cameroon is perennial with the intensity increasing with the prevalence of parasitaemia and amount of rainfall. The human population is therefore constantly exposed to infective mosquito bites that result in the development and maintenance of naturally acquired immunity [31]. As shown, an individual living in Tiko, Limbe and Idenau will on the average receive 287, 160 and 149 infective mosquito bites/year respectively. The prevalence of malaria parasitaemia was inversely related to age. The absence of malaria parasites in persons older than 15 years in Idenau is paradoxical, and may not be fully justified since only 11 individuals belonging to this age group accepted to be part of the study in this locality. The fact that gametocytes were found mainly in children less than five years of age suggests this age group as the major parasite reservoir for malaria vectors. Compared to the high CSA rates obtained, the gametocyte index was unexpectedly low. It is suggested that many of the participants might have been exposed to antimalarial drugs prior to the survey that resulted in the low gametocyte index.

*Anopheles gambiae *s.s. M molecular form, *An. funestus *and *An. nili *were the main malaria vectors in the surveyed areas with *An. gambiae *being predominant and the most aggressive. This observation in Tiko, a rural settlement, is different from that in the rural areas of southern-forested Cameroon where *An. gambiae *has been reported not to be the major malaria vector [32,33]. *Anopheles nili *was conspicuously present only in Tiko. *Anopheles funestus *was present in all three localities in low numbers, but generally had higher infection rates. This is due to its high anthropophily, which explains its efficiency in malaria transmission [17]. Vector abundance varied seasonally and increased with increasing rainfall, resulting in the proliferation of *An. gambiae *populations. The presence of *An. rufipes*, a better known zoophilic species [17], and other non malaria vectors is indicative of the level of nuisance that inhabitants of these areas get from these mosquitoes.

Interestingly, *An. melas *was absent amongst the human biting populations of the *An. gambiae *complex but occurred in the samples reared from wild larvae from Limbe and Idenau. Unlike in many areas along the coast of West Africa where it is reported to be an important malaria vector [34-36], *An. melas *appears not to play a role in malaria transmission in coastal Cameroon.

*Anopheles melas *was identified from breeding sources within 2 km from the coastline in Idenau and within 3 to 5 km from the coastline in Limbe. Its absence in Tiko may be due to several reasons ranging from human, climatic and ecological determinants such as reduction in the salt content or complete absence of saline water as distance from the coastline increases. With rapid growth in population, urbanization and agro-industrial activities within the CDC, this area has been deprived of its mangrove swamps that are preferential for *An. melas *breeding [36].

The observed parity rates were high. This indicates that older populations of mosquitoes tend to accumulate with time. This allows for increased feeding frequencies and thus, increased chances of the vectors becoming infected or even re-infected during subsequent feeding [37].

Although the infection rates (CSA rates) for *An. gambiae *showed high susceptibility to *P. falciparum *infection, the mean CSA rates for *An. funestus *were generally higher despite the small numbers. This is due to the marked affinity *An. funestus *has to humans and their dwellings, thus increasing their chances of becoming infected [38]. This does not, however, underestimate the role of *An. nili *having relatively high infection rates in this area. To minimize false positive CSA ELISA, only the heads and thoraxes were used and the presence of sporozoites was confirmed by PCR [27,28,39]. Thus, CSA rates were calculated using only specimens confirmed positive by PCR. The ability of *An. gambiae *to support the development and propagation of more than one parasite species at the same time is indicative that it can transmit more than one parasite species simultaneously, as earlier demonstrated in other areas of Africa [40,41]. The sporozoite densities in this study were lower than was reported in western Kenya and in Tanzania, implying that the number of heavy infections were smaller than the number of light infections [42,43].

Malaria transmission dynamics have been shown to vary greatly across Africa [14]. Mean annual inoculation rates vary from less than one to over 1,000 ib/p/y [44,45]. The observed mean annual inoculation rates in Tiko, Limbe and Idenau are therefore high and corroborate previous studies in other parts of Cameroon [5,33,46-48]. Despite the higher infection rates observed for *An. funestus*, the EIR for *An. gambiae *was generally higher. This is probably due to a multiplier effect of the high density and biting rate of *An. gambiae*, an observation similar to that in coastal Tanzania [42].

## Conclusion

Transmission in coastal Cameroon occurs perennially with the periodicity of heightened entomological inoculation rates coinciding with periods of increased vector density, biting rates, prevalence of malaria parasitaemia in the indigenous population and rainfall. *Anopheles melas*, apart from constituting only a minor proportion of the *An. gambiae *complex, was not anthropophagic, and therefore not an important vector in the surveyed areas. The findings provide a baseline for evidence-based planning and implementation of malaria control activities.

## Authors' contributions

JDB: Conception of study and experimental design, data collection, analysis and interpretation, preparation of the manuscript. LM: Contributed substantially to conception, study design, interpretation of data, and critically reading the manuscript for important intellectual content. VPKT: Contributed to designing and interpretation of the data as well as critically revising the manuscript. MC: Experimental design of the study and critically revised manuscript for intellectual content. RGFL: Participation in study design and coordination. Interpretation of the data, critically reading of the manuscript. All authors read and approved the manuscript.
